# Immunotherapy for Chordoma and Chondrosarcoma: Current Evidence

**DOI:** 10.3390/cancers13102408

**Published:** 2021-05-17

**Authors:** Jeffrey I. Traylor, Mark N. Pernik, Aaron R. Plitt, Michael Lim, Tomas Garzon-Muvdi

**Affiliations:** 1 Department of Neurological Surgery, The University of Texas Southwestern Medical Center, Dallas, TX 75235, USA; jeffrey.traylor@utsouthwestern.edu (J.I.T.); mark.pernik@utsouthwestern.edu (M.N.P.); aaron.plitt@phhs.org (A.R.P.); 2 Department of Neurosurgery, Stanford University Medical Center, Stanford, CA 94305, USA; mklim@stanford.edu

**Keywords:** chordoma, chondrosarcoma, brachyury, PD-1, immunotherapy

## Abstract

**Simple Summary:**

Chordomas and chondrosarcomas are rare tumors that can occur within the skull base and spinal column and are often resistant to chemotherapy and radiation. While surgical removal of these tumors is helpful, residual tumors that could not be removed surgically can often lead to recurrences. Recent advances have revealed that chordomas and chondrosarcomas have many interactions with our host immune system that may drive the progression of these tumors. In our paper, we discuss these recent advances, potential treatment targets that leverage the immune interactions, and emerging clinical data.

**Abstract:**

Chordomas and chondrosarcomas are rare but devastating neoplasms that are characterized by chemoradiation resistance. For both tumors, surgical resection is the cornerstone of management. Immunotherapy agents are increasingly improving outcomes in multiple cancer subtypes and are being explored in chordoma and chondrosarcoma alike. In chordoma, brachyury has been identified as a prominent biomarker and potential molecular immunotherapy target as well as PD-1 inhibition. While studies on immunotherapy in chondrosarcoma are sparse, there is emerging evidence and ongoing clinical trials for PD-1 as well as IDH inhibitors. This review highlights potential biomarkers and targets for immunotherapy in chordoma and chondrosarcoma, as well as current clinical evidence and ongoing trials.

## 1. Introduction

Chordomas are rare, malignant neoplasms that arise from vestigial notochord remnants. Although these tumors may arise anywhere along the spinal column, they have a predilection for the clivus and sacrococcygeal area, although they can also be found throughout the spine [1]. Surgical resection is the mainstay of therapy for these patients; however, deep-seated or locally advanced tumors preclude gross-total resection due to anatomical constraints. Chordoma is generally resistant to chemotherapy, and the standard of care involves proton-beam radiotherapy or high dose photon radiotherapy to the resection cavity [2,3]. Despite maximal safe resection and radiation, chordoma displays high rates (>50%) of loco-regional recurrence [4], and some patients experience distant metastatic disease [5]. Further, there are currently no chemo or immunotherapies approved for the treatment of chordoma, and median survival is approximately 5–8 years from the time of diagnosis with 5-year survival rates around 67% [6,7,8,9]. Effective adjuvant therapies are thus desperately needed to improve the prognosis in this patient population. Pharmacotherapy development has been hindered in the past, primarily due to the ‘quiet’ chordoma genome—that has few known drivers of disease from which to develop targeted therapies [10]. Nevertheless, research advances in the last decade have identified biomarkers and potential therapeutic targets, of which several have been developed and are undergoing clinical trials [11].

Chondrosarcoma of the skull base, though histopathologically distinct, has similar radiologic features to chordoma as well as a similar clinical presentation. Arising from embryonal endochondral cells of the skull base, chondrosarcoma confers a better prognosis than chordoma of the same region, with a median projected survival of 22 years [12]. The majority of these tumors are low to intermediate grade; however, as many as 10% of these tumors exhibit high-grade features, including distant metastases, and confer a poor overall survival rate [13]. Both chordoma and chondrosarcoma produce an abundant extracellular matrix, which contributes to the histologic characterization of these tumors. Like chordoma, chondrosarcoma is largely unresponsive to most chemotherapies [14]; however, some studies have reported a response to radiation, with significantly lower 5-year recurrence rates with adjuvant radiotherapy [15]. Histologic variants of chondrosarcoma have also been shown to contribute to specific clinical characteristics, including dedifferentiated, mesenchymal, and clear cell subtypes [16]. These subtypes have been shown to have specific genetic alterations including mutations in COL2A1, IDH, and the hedgehog signaling pathway [17].

## 2. Chordoma Immune Microenvironment

**Figure 1:**
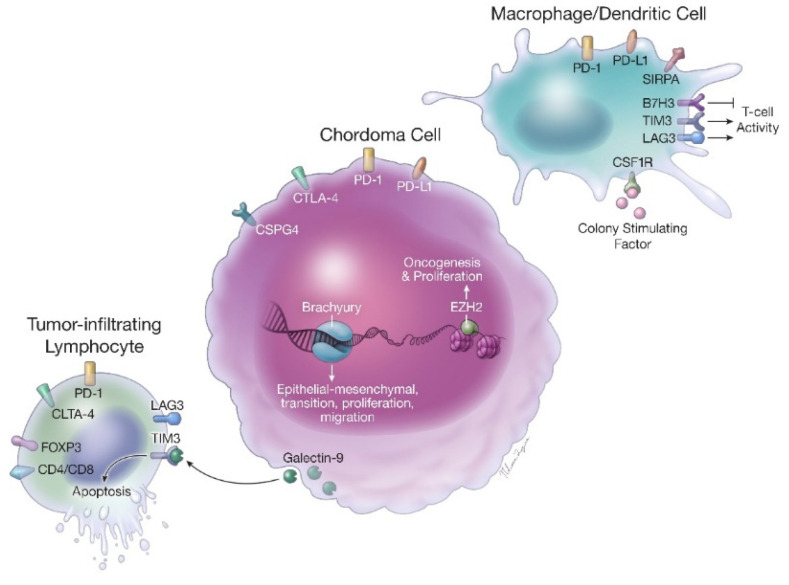
The chordoma immune microenvironment.

Chordomas have extensive interactions with the immune system that may help to predict tumor aggressiveness, responses to existing therapies and the development of novel treatments (Figure 1). Zou and colleagues have studied this interaction extensively, specifically in regard to the PD-1/PDL-1 pathway [18,19,20,21]. In their initial investigations, they reported that the expression of PD-L1 in tumor tissue was associated with advanced chordoma stages and higher levels of tumor-infiltrating lymphocytes (TIL), whereas PD-L1 expression on TIL was associated with improved local recurrence free survival (LRFS) and overall survival [18,19]. Conversely, TIL expression of PD-1 was associated with worse LRFS and overall survival [18,19]. Notably, both Zou et al. and others have found that a significant subset of chordomas express PD-L1/PD-1; however, this is likely driven by TILs and macrophages as opposed to expression by tumoral tissue, which seems to be less common [18,19,22,23]. PD-1/PD-L1 signaling may also be regulated by microRNA in chordomas, with lower expression of miR-574-3p and higher PD-L1 expression being associated with worse overall survival [19]. These data indicate that the locally aggressive nature of chordoma may be driven in part by immune interactions within the tumor microenvironment, allowing immune evasion and tumor progression.

In addition to the PD-1/PD-L1 pathway, other interactions between chordoma and the immune system have been elucidated. Chordomas positive for Galectin-9 (Gal9), a molecule that interacts with TIM3^+^ T-cells to induce apoptosis, had greater local invasiveness and lower Karnofsky performance status scores [24]. Higher TIM3^+^ TILs were also associated with invasiveness and lower performance status scores [24]. Gal9 expression, in turn, may also be regulated by microRNA levels (miR-455-5p), with downregulation of miR-455-5p being predictive of chordoma invasiveness and prognosis [24]. CTLA-4 is also expressed in a significant portion of chordomas, both on TIL and tumoral tissue [25]. Higher expression of CTLA-4 on chordoma tumor tissue was associated with decreased disease-free survival and overall survival, and higher CTLA-4 expression on TIL was associated with decreased disease-free survival only [25].

A few authors have proposed immune-based scoring systems for chordomas to help to predict tumor aggressiveness and outcomes [19,21,26]. One study found that in their proposed scoring system of cells by levels of CD3^+^ and CD8^+^ TIL in the tumor interior, higher levels of TILs were associated with increased LRFS and overall survival [18]. Another study trained a scoring model which identified the presence of FOXP3 and PD-1 in tumor tissue, as well as stromal FOXP3 and CD8 infiltration as predictors of tumor outcomes, with their model effectively differentiating disparate tumor outcomes within the same Enneking stage [21]. A third study found that higher platelet-to-lymphocyte and neutrophil-to-lymphocyte ratios were associated with worse overall survival [26]. This may indicate that a non-specific inflammatory response, as opposed to a targeted immune response driven by lymphocytes, may be an indicator of worse prognosis [26,27]. These studies make apparent that TIL, as well as systemic inflammation, may contribute to chordoma outcomes [26,27]. Future work should focus on whether these scoring systems have any implications or serve as biomarkers for the success of immune-based therapies for chordomas.

## 3. Chordoma Biomarkers

Several biomarkers have been described in chordomas that contribute to tumor progression. While biomarkers such as brachyury, a transcription factor that is important for chordoma progression, have been robustly investigated and now have associated immunotherapeutic agents being tested in trials, other biomarkers such as CSPG4 require additional research to elucidate potential clinical relevance. In this section, we review the known biomarkers of chordoma, their roles in chordoma progression, and their interactions with the chordoma immune microenvironment.

### 3.1. CSPG4

In one study, 62% of chordomas expressed a high molecular weight-melanoma associated antigen (HMW-MAA), an antigen discovered in melanoma for which an antagonistic immunotherapy already exists [28,29,30]. HMW-MAA, also known as CSPG4, is detected in a majority of chordomas and chondrosarcomas and was implicated as an immunotherapy target in both tumors due to its function in cellular migration, survival, and invasion [30]. A study by Schoenfeld and colleagues found that CSPG4 expression doubled the risk of death and increased the risk of metastatic disease in chordoma [31].

### 3.2. Brachyury

A T-box-family transcription factor expressed by the T or TBXT gene, brachyury has been shown to be essential in regulating notochord development [32]. Moreover, overexpression of brachyury is unique to chordoma when compared to other neoplasms [33]. Subsequent studies have further elucidated brachyury as a driver of disease with T gene duplication and single-nucleotide variations reported as contributing to chordoma development [34,35]. Although brachyury has been identified as oncogenic and a biomarker in chordoma, the mechanisms for dysregulation are not yet understood. Sharifnia et al. utilized CRISPR-Cas9 screening to identify brachyury dependencies and therapeutic targets in chordoma [36]. Specifically, the authors described the targeting of brachyury transcription factor addiction, which describes a reliance on specific oncogenes for tumor progression as a strategy for therapeutic development in chordoma [36,37]. They reported selectively lethal single-guide RNAs targeting the *T* gene against chordoma, underscoring brachyury as a potential pharmacologic target [36]. The authors also performed a screen of several small molecules on chordoma cell lines. Of those tested, 28 antiproliferative compounds were identified [36]. Specifically, inhibition of CDK7/12/13 and CDK9 transcription reduced brachyury expression and suppressed proliferation of chordoma cell lines [36]. Although brachyury has been called an ‘intractable’ therapeutic target, afatanib has been shown to promote degradation of brachyury and EGFR in chordoma cell lines [38,39]. Interestingly, brachyury may also be targeted by the immune system. In an open label phase I trial of seven patients with chordoma immunized to brachyury, 43% (3/7) of patients developed a T-cell brachyury-specific response; however outcome data were unavailable [40].

### 3.3. Tumor–Stroma Ratio

Diagnosis and therapeutic regimens were conventionally identified and selected based on histopathological analysis and tumor cell characteristics. However, an increasing number of studies have investigated characteristics of tumor-associated stroma in relation to neoplastic cells. The tumor–stroma ratio (TSR), representing the percentage of tumor cells relative to stroma cells, has been explored as a significant prognostic factor in several malignancies, including non-small cell lung cancer, breast cancer, esophageal cancer, and cervical cancer [41]. Specifically, a high proportion of stroma in cancer tissue has been associated with worse clinical outcomes in patients with solid tumors. One study to date reported on the predictive value of TSR in spinal chordoma, specifically, and found that low TSR independently predicted poor overall survival and loco-regional recurrence [42]. Further, stromal involvement negatively correlated with PD-L1 expression as well as TIL density [23].

### 3.4. CTLA-4

One study has identified that CTLA-4 is expressed in a significant portion of chordomas [25]. He et al. investigated the rates of CTLA-4 expression in chordoma and TILs and found that higher expression conferred a significantly shorter continuous disease-free survival and overall survival [25].

### 3.5. EZH2

Enhancer of zeste homolog 2 (EZH2) is a histone methyltransferase that regulates cellular differentiation and plays an important physiologic role in embryogenesis. Activating mutations of EZH2 have been shown to lead to oncogenic transformation and proliferative dependency on EZH2 activity in cancer [43]. Further, mutations of the INI1 (SMARCB1) subunit of the switch/sucrose non-fermentable complex (SWI/SNF) have been thought to lead to EZH2-dependent tumor formation [44]. Two small studies reported a lack of nuclear expression of INI1 (SMARCB1) in poorly differentiated chordoma [45,46].

## 4. Chondrosarcoma Immune Microenvironment

The immune microenvironment of chondrosarcoma is poorly understood. In order to determine whether checkpoint blockade or other types of immunotherapy are effective against chondrosarcoma, a thorough understanding of the immune milieu is needed. Several microenvironmental mechanisms have been theorized to contribute to chemoresistance—specifically, the expression of membrane-bound P-glycoprotein [47]. Chondrosarcomas have demonstrated CD163^+^ macrophage infiltration, which has been associated with more invasive and higher-grade chondrosarcomas, while higher concentrations of CD8^+^ T cells have shown to repress chondrosarcoma progression [48]. A 2016 study by Kostine et al. reported that almost half of dedifferentiated chondrosarcomas expressed PD-L1, which correlated with high concentrations of TIL and HLA class I expression [49].

A 2020 study by Iseulys and colleagues found that tumor-associated macrophages were the predominant immune cell type in the immune environment of chondrosarcoma [50]. They also found that high levels of CD68^+^ macrophages were associated with metastatic disease at diagnosis and a poor prognosis. The authors also reported increased expression of the colony-stimulating factor 1 receptor (CSF1R), signal regulatory protein alpha (SIRPA), B7 superfamily member-H3 (B7H3), T cell immunoglobulin mucin (TIM3) and lymphocyte activation gene-3 (LAG3). CSF1R is a crucial signaling mechanism for differentiation and survival in macrophages [51]. Also expressed by macrophages, SIRPA is activated by the presence of CD47 on normal and tumor cells to prevent phagocytosis [52]. B7H3 is a membrane protein expressed by antigen-presenting cells (APC) that inhibits T cell activity, but it is also suspected to play an important role in migration, invasion, and angiogenesis in several malignancies [53]. TIM3 is a transmembrane protein expressed by CD4^+^ and CD8^+^ T cells that has been shown to influence macrophage activation in the context of autoimmune diseases and is a marker of T cell exhaustion [54,55]. LAG3 is a membrane-bound, protein immune-checkpoint receptor related to CD4 on T cells that serves to regulate T cell activation and proliferation [56].

## 5. Chondrosarcoma Biomarkers

While chondrosarcomas have a similar presentation and anatomical distribution to chordomas, they also have unique biomarkers that regulate tumor progression and survival. Due to the rarity of chondrosarcomas in comparison to chordomas, the molecular underpinnings of chondrosarcoma are not as well understood. In this section, we highlight key biomarkers that may contribute to the chondrosarcoma progression that may serve as potential immunotherapeutic targets.

### 5.1. Aurora Kinase

The aurora kinases belong to the family of serine and threonine kinases, which have a role in regulating the cell cycle through control of centriole and microtubule function [57]. Dysregulation of aurora kinases A and B has further been reported to promote tumorigenesis, and they are highly expressed in several malignant tumors [57]. A 2012 study by Liang and colleagues demonstrated that aurora kinases A and B were highly expressed in higher-grade chondrosarcoma when compared to lower grade chondrosarcoma [58]. Further, the authors reported a significantly reduced survival in patients with aurora kinase A expression and thus concluded that it was an independent marker of poor prognosis.

### 5.2. Hypoxia Inducible Factor and Beclin-1

Hypoxia inducible factor (HIF) is a key transcription factor in the cellular response to hypoxic conditions and has been implicated in tumor survival in ischemic conditions [59]. A 2011 study identified that HIF-1α as well as Bcl-xL expression was significantly higher in chondrosarcomas when compared to benign cartilaginous tumors and conferred a significantly worse overall survival [60,61]. Beclin-1 is a mediator of autophagy that is downregulated in hypoxic conditions, delaying apoptosis [62]. Its downregulation has been linked to significant compromises in appropriate autophagic responses to hypoxia in several malignancies, including breast, ovarian, and colorectal [63,64,65], as well as high-grade glial neoplasms [66]. A 2011 study by Chen et al. identified a significant inverse relationship between beclin-1 and HIF-2α [60]. They also reported that high HIF-2α expression and negative beclin-1 levels were significant predictors for poor overall survival, making the expression of HIF-1α and HIF-2α important biomarkers in chondrosarcoma [60].

### 5.3. Isocitrate Dehydrogenase

Somatic mutations of isocitrate dehydrogenase (IDH) 1 and 2 have been established as biomarkers in glioma and acute myeloid leukemia [67,68]. Amary et al. identified IDH1 and IDH2 mutations in approximately half of chondromas and chondrosarcomas studied [69] and in another publication reported that almost all cases of Ollier disease and Mafucci syndrome (both characterized by multiple cartilaginous tumors) were associated with IDH mutations [70]. Preclinical evidence is sparse and conflicting for the inhibition of IDH in chondrosarcoma cells. One study reported suppressed tumorigenic activity and reduced 2-hydroxyglutarate in cell lines [71], while another publication of the same year reported decreased 2-hydroxyglutarate without significant effects on tumorigenic properties in chondrosarcoma cell lines [72].

### 5.4. Plasminogen Activator Inhibitor 1

Plasminogen activator inhibitor 1 (PAI-1) is an enzyme that prevents the conversion of plasminogen to plasmin by inhibition of urokinase plasminogen activator. A 2009 study by Rozeman et al. reported that increased PAI-1 conferred a significantly better overall prognosis [73].

### 5.5. Hedgehog Signaling

The Hedgehog signaling pathway has an important role in regulating cell proliferation and differentiation during embryogenesis. Hedgehog signaling is essential for chondrocyte differentiation; additionally, chondrosarcomas express high levels of the Hedgehog target genes GLI1 and PTCH1 and, when constitutively activated, upregulated tumor cell proliferation [74].

### 5.6. mTOR

The mechanistic target of rapamycin (mTOR) pathway plays a critical role in tumor survival, metabolism, and proliferation [75]. Inhibition of mTOR in chondrosarcoma cell lines and animal models has led to anti-tumor responses via reduction of glycolysis, oxidative metabolism, cellular proliferation, and Glut1 and HIF-1α expression [76,77].

## 6. Preclinical and Clinical Studies

### 6.1. Chordoma

The first report of any clinical response of chordoma to immunotherapy was in 2017. Three chordoma patients with failure of standard therapies were treated with tumor-based vaccine or checkpoint inhibitor therapy [78]. In the two cases of treatment-resistant chordomas, immune checkpoint inhibitors pembrolizumab and nivolumab were used as a last resort [78]. In the patient with a C3 conventional chordoma treated with pembrolizumab, there was marked radiographic tumor regression and recovery of a facial palsy that was sustained at the 6-month follow-up period [78]. The second patient with a petro-clival chordoma treated with nivolumab also had radiographic and clinical improvement for 9 months, followed by tumor progression [78]. The third patient in this series with a locally aggressive clival chondroid chordoma was treated with MVX-ONCO-1 through an ongoing clinical trial (ClinicalTrials.gov (accessed on 2 March 2021) Identifier: NCT02193503): a personalized anti-tumor vaccine consisting of irradiated individual tumor cells, surrounded by a capsule of allogeneic, genetically modified cells that secrete GM-CSF to promote antigen-presenting cells to tumor neoantigens [78,79,80]. At 19 months after treatment, this patient has had sustained radiographic improvement; the implications of which are difficult to determine due to the lack of published Phase I clinical trial results of MVX-ONCO-1, which is being tested on metastatic carcinoma or refractory sarcoma [78]. Brachyury immunohistochemistry staining was initially positive in all three cases, but negative after de-differentiation and, in one case, prior to commencement of checkpoint inhibitor therapy [78]. Another case report described a patient with metastatic chordoma who, after initiation of pembrolizumab, had a reduction in metastatic burden by >30% and progression-free survival for over 9 months until progression occurred and pembrolizumab was discontinued [81]. These case series indicate that there are select chordomas that do respond to immunotherapy. However, it is not clear what contributed to their responses—tumor genetics, immune microenvironment, tumor checkpoint inhibitor expression, or otherwise [78]. In one study on patient-derived chordoma organoids, expression of PD-L1 in chordoma was correlated with TILs but did not predict response to immune checkpoint inhibitor therapy [82]. Similarly, a 2016 study of the anti-PD-L1 antibody avelumab was investigated in four chordoma cell lines and demonstrated antibody-mediated cell cytotoxicity, particularly when co-incubated with brachyury-specific CD8^+^ T cells [83].

In 2013, Hamilton and colleagues investigated the efficacy of a newly developed brachyury-yeast vaccine (GI-6301) and found that it elicited a brachyury-specific CD4^+^ and CD8^+^ T-cell response in vitro [84]. A follow-up phase I trial of 34 patients demonstrated safety and brachyury-specific T-cell response in the majority of the cohort [85]. A phase II trial was developed but stopped early due to no difference in response between GI-1301 and placebo [86]. An ongoing phase II trial, however, is currently testing a transgenic BN-brachyury vaccine in chordoma undergoing radiation therapy (NCT03595228).

Inhibition of EZH2 with tazemetostat has also been explored [87]. A phase I study of tazemetostat, an inhibitor of EZH2, showed antitumor activity in patients with various lymphomas and sarcomas [88]. In a case report of a patient with metastatic chordoma who was treated with tazemetostat, treatment resulted in a >2-year response characterized by tumor-infiltrating T-lymphocytes and checkpoint activation [87]. This patient was enrolled in the ongoing Phase II clinical trial of tazemetostat for INI1-negative tumors or refractory sarcomas (ClinicalTrials.gov (accessed on 2 March 2021) Identifier NCT02601950).

Owing to the rarity of chordomas, literature on their response to immunotherapy is scarce. Predicting which chordomas will respond to immunotherapy is not possible with current evidence. Additional clinicopathological studies are needed to elucidate cellular markers. There are two ongoing clinical trials testing nivolumab (anti-PD-1) for advanced chordomas (ClinicalTrials.gov (accessed on 2 March 2021) Identifier NCT02989636 [89] and NCT03623854 [90], respectively).

### 6.2. Chondrosarcoma

Approximately 50% of dedifferentiated chondrosarcomas have been reported to express PD-L1 [49], which has given cause for investigation of anti-PD-1 and/or PD-L1 antibodies in this patient population. Despite the emerging preclinical evidence, clinical evidence remains limited, with a number of clinical trials currently ongoing (Table 1). A 2016 study by Paoluzzi et al. retrospectively analyzed the response of nivolumab, an anti-PD1 monoclonal antibody, in patients with metastatic sarcoma, only two of whom had a diagnosis of chondrosarcoma [91]. The authors reported a partial response after six cycles of nivolumab alone in a patient with dedifferentiated chondrosarcoma, while stable disease was observed in a patient with mesenchymal chondrosarcoma after four cycles of nivolumab. Another case report of a 67-year-old man with metastatic chondrosarcoma, treated with nivolumab, reported near complete response in pulmonary nodules after four cycles [92]. The SARC028 trial, a multicenter phase II trial investigating the activity of pembrolizumab in patients with advanced soft-tissue and bone sarcoma, reported a partial objective response in only one of five patients with chondrosarcoma [93]. There is also an ongoing phase II trial investigating the efficacy of nivolumab plus ipilimumab in non-resectable sarcoma and endometrial carcinoma (ClinicalTrials.gov (accessed on 2 March 2021) Identifier: NCT02982486) [94]. Another ongoing phase I/II study is investigating combined nivolumab and the mTOR inhibitor ABI-009 in patients with chondrosarcoma and other advanced malignancies (ClinicalTrials.gov (accessed on 2 March 2021) Identifier: NCT03190174) [95].

There are currently three ongoing clinical trials investigating IDH inhibition in chondrosarcoma patients. The first is a phase II trial of AG-120 in patients with glioma, cholangiocarcinoma, chondrosarcoma, and other advanced solid tumors (ClinicalTrials.gov (accessed on 2 March 2021) Identifier: NCT04278781) [96]. The second is a phase I study of BAY1436032 in patients with IDH mutated solid tumors (ClinicalTrials.gov (accessed on 2 March 2021) Identifier: NCT02746081) [97]. Finally, a phase I/II study of AG-221 is underway for patients with chondrosarcoma as well as other solid tumors and T-cell lymphoma (ClinicalTrials.gov (accessed on 2 March 2021) Identifier: NCT02273739) [98].

In light of the results demonstrating the importance of the hedgehog signaling pathway in chondrosarcoma, a phase II study is underway investigating the efficacy of vismodegib, a Hedgehog signaling pathway inhibitor, in patients with advanced chondrosarcoma (ClinicalTrials.gov (accessed on 2 March 2021) Identifier: NCT01267955) [99].

## 7. Conclusions

Chordoma and chondrosarcoma are malignancies that can arise from the skull base and spine that present a particular challenge to manage due to associated resistance to conventional therapies such as chemoradiation. Several studies in the last decade have detailed the immune microenvironment as well as genetic and molecular biomarkers in these cancers, which have laid the foundation for individualized therapy and immunotherapeutic targets that show promise for improving outcomes in these patients. This is exemplified in chordoma with the transcription factor brachyury, which is now a treatment target of multiple ongoing clinical trials that leverage the immune system to target brachyury. Chordoma and chondrosarcoma have also benefited from the robust investigation of checkpoint inhibitors for solid tumors, with numerous promising case reports and ongoing trials using these immunotherapeutic agents. While additional preclinical and clinical data are needed, available evidence indicates that there are significant interactions between chordoma and chondrosarcoma and the immune system, particularly tumor-infiltrating lymphocytes. Several biomarkers exist that have not been studied in-depth and warrant detailed investigation to elucidate their clinical relevance and potential as treatment targets. Targeted therapies such as immunotherapy may have a robust impact for select patients with chordoma and chondrosarcoma.

## Figures and Tables

**Table 1:** Ongoing chordoma and chondrosarcoma immunotherapy clinical trials.

Study Title	Drug(s)	Phase	Population	Estimated Enrollment	Study Sites	Primary Completion Date	Trial Registration Number
Nivolumab with or without Stereotactic Radiosurgery in Treating Patients with Recurrent, Advanced, or Metastatic Chordoma	Nivolumab ± stereotactic radiosurgery	Phase I	Recurrent or metastatic chordomas	33	Johns Hopkins University, Memorial Sloan-Kettering Cancer Center	March 2022	NCT02989636
Nivolumab and Relatlimab in Treating Participants with Advanced Chordoma	Nivolumab + relatlimab	Phase II	Metastatic or locally advanced/ unresectable chordomas	20	University of California, Los Angeles	April 2021	NCT03623854
BN-Brachyury and Radiation in Chordoma	BN-Brachyury (transgenic vaccine)	Phase II	Chordoma undergoing radiation therapy	29	Mayo Clinic (AZ, FL), Massachusetts General Hospital, Washington University, MD Anderson	April 2022	NCT03595228
QUILT-3.011 Phase 2 Yeast-Brachyury Vaccine Chordoma	Yeast-brachyury vaccine	Phase II	Unresectable chordoma with planned radiation therapy	55	NIH Clinical Center	March 2020	NCT02383498
QUILT-3.091 NANT Chordoma Vaccine vs. Radiation in Subjects with Unresectable Chordoma	NANT chordoma vaccine (brachyury immunogenic)	Phase I & II	Unresectable chordoma	N/A	Chan Soon-Shiong Institute for Medicine	August 2022	NCT03647423
Talimogene Laherparepvec, Nivolumab and Trabectedin for Sarcoma (TNT)	Talimogene laherparepvec, nivolumab, and trabectedin as first, second- or third-line therapy	Phase II	Locally advanced unresectable or metastatic sarcoma including desmoid tumor or chordoma	40	Sarcoma Oncology Center	December 2022	NCT03886311
MVX-ONCO-1 in Patients with Solid Tumor	MVX-ONCO-1 vaccine	Phase I	Advanced metastatic carcinoma or sarcoma refractory to all treatments	35	Hopitaux Universitaires de Genève	December 2021	NCT02193503
A Phase II, Multicenter Study of the EZH2 Inhibitor Tazemetostat in Adult Subjects with INI1-Negative Tumors or Relapsed/Refractory Synovial Sarcoma	Tazemetostat	Phase II	INI1-negative malignancy with exhausted therapies (including chordoma and chondrosarcoma)	250	32 international study locations	May 2023	NCT02601950
A Phase II of Nivolumab Plus Ipilimumab in Non-resectable Sarcoma and Endometrial Carcinoma	Nivolumab + ipilimumab	Phase II	Nonresectable/ metastatic sarcoma (including chondrosarcoma) or endometrial carcinoma	60	Assaf-Harofeh Medical Center	December 2020	NCT02982486
Nivolumab (Opdivo^®^) Plus ABI-009 (Nab-rapamycin) for Advanced Sarcoma and Certain Cancers	Nivolumab + ABI-009	Phase I & II	Metastatic or locally advanced (and nonresectable) sarcomas and other cancers (including chordoma)	40	Sarcoma Oncology Research Center	April 2021	NCT03190174
AG-120 in People with IDH1 Mutant Chondrosarcoma	AG-120 (IDH inhibitor)	Phase II	Locally advanced or metastatic or recurrent operable chondrosarcoma with IDH1 gene mutation	17	Memorial Sloan-Kettering Cancer Center, MD Anderson Cancer Center	March 2023	NCT04278781
Phase I Study of BAY1436032 in IDH1-mutant Advanced Solid Tumors	BAY1436032 (IDH inhibitor)	Phase I	Any IDH1-R132X-mutant solid tumor	81	Institutions N/A	March 2021	NCT02746081
Vismodegib in Treating Patients with Advanced Chondrosarcomas	Vismodegib	Phase II	Confirmed chondrosarcoma	45	Multiple French institutions	June 2018	NCT01267955

## Abbreviations

TIL	Tumor-infiltrating lymphocytes
LRFS	Local recurrence free survival
TSR	Tumor-stroma ratio
CSF1R	Colony-stimulating factor 1 receptor
SIRPA	Signal regulatory protein alpha
B7H3	B7 superfamily member-H3
TIM3	T cell immunoglobulin mucin
LAG3	Lymphocyte activation gene 3
HIF-2α	Hypoxia inducible factor-2α
IDH	Isocitrate dehydrogenase
PAI-1	Plasminogen activator inhibitor 1
EZH2	Enhancer of zeste homolog 2
SWI/SNF	Switch/sucrose non-fermentable complex

## References

[1] Flanagan A.M., Yamaguchi T. (2013). World Health Organization. Classification of Tumours of Soft Tissue and Bone: WHO Classification of Tumours.

[2] Zhou J., Sun J., Bai H.X., Huang X., Zou Y., Tan X., Zhang Z., Tang X., Tao Y., Xiao B. (2017). Prognostic factors in patients with spinal chordoma: An integrative analysis of 682 patients. Clin. Neurosurg..

[3] Holliday E.B., Mitra H.S., Somerson J.S., Rhines L.D., Mahajan A., Brown P.D., Grosshans D.R. (2015). postoperative proton therapy for chordomas and chondrosarcomas of the spine. Spine.

[4] Stacchiotti S., Gronchi A., Fossati P., Akiyama T., Alapetite C., Baumann M., Blay J.Y., Bolle S., Boriani S., Bruzzi P. (2017). Best practices for the management of local-regional recurrent chordoma: A position paper by the Chordoma Global Consensus Group. Ann. Oncol..

[5] Baratti D., Gronchi A., Pennacchioli E., Lozza L., Colecchia M., Fiore M., Santinami M. (2003). Chordoma: Natural history and results in 28 patients treated at a single institution. Ann. Surg Oncol..

[6] Jones P.S., Aghi M.K., Muzikansky A., Shih H.A., Barker F.G., Curry W.T. (2014). Outcomes and patterns of care in adult skull base chordomas from the Surveillance, Epidemiology, and End Results (SEER) database. J. Clin. Neurosci..

[7] Boari N., Gagliardi F., Cavalli A., Gemma M., Ferrari L., Riva P., Mortini P. (2016). Skull base chordomas: Clinical outcome in a consecutive series of 45 patients with long-term follow-up and evaluation of clinical and biological prognostic factors. J. Neurosurg..

[8] Bohman L.-E., Koch M., Bailey R.L., Alonso-Basanta M., Lee J.Y. (2014). Skull base chordoma and chondrosarcoma: Influence of clinical and demographic factors on prognosis: A SEER analysis. World Neurosurg..

[9] Pan Y., Lu L., Chen J., Zhong Y., Dai Z. (2018). Analysis of prognostic factors for survival in patients with primary spinal chordoma using the SEER Registry from 1973 to 2014. J. Orthop. Surg. Res..

[10] Tarpey P.S., Behjati S., Young M.D., Martincorena I., Alexandrov L.B., Farndon S.J., Guzzo C., Hardy C., Latimer C., Butler A.P. (2017). The driver landscape of sporadic chordoma. Nat. Commun..

[11] Gill C.M., Fowkes M., Shrivastava R. (2020). K Emerging therapeutic targets in chordomas: A review of the literature in the genomic era. Clin. Neurosurg..

[12] Jones P.S., Aghi M.K., Muzikansky A., Shih H.A., Barker F.G., Curry W.T. (2014). Outcomes and patterns of care in adult skull base chondrosarcomas from the SEER database. J. Clin. Neurosci..

[13] Folkert I.W., Devalaraja S., Linette G.P., Weber K., Haldar M. (2019). Primary bone tumors: Challenges and opportunities for CAR-T therapies. J. Bone Miner. Res..

[14] Riedel R.F., Larrier N., Dodd L., Kirsch D., Martinez S., Brigman B.E. (2009). The clinical management of chondrosarcoma. Curr. Treat. Options Oncol..

[15] Bloch O.G., Jian B.J., Yang I., Han S.J., Aranda D., Ahn B.J., Parsa A.T. (2010). Cranial chondrosarcoma and recurrence. Skull Base.

[16] Chebib I., Hornicek F.J., Bredella M.A., Deshpande V., Nielsen G.P. (2014). Histologic variants of chondrosarcoma. Diagn. Histopathol..

[17] Chow W. (2018). Chondrosarcoma: Biology, genetics, and epigenetics. F1000Research.

[18] Zou M.-X., Peng A.B., Lv G.-H., Wang X.B., Li J., She X.-L., Jiang Y. (2016). Expression of programmed death-1 ligand (PD-L1) in tumor-infiltrating lymphocytes is associated with favorable spinal chordoma prognosis. Am. J. Transl. Res..

[19] Zou M.-X., Guo K.-M., Lv G.-H., Huang W., Xiao-Ling S., Wang X.-B., Jiang Y., She X.-L. (2018). Clinicopathologic implications of CD8+/Foxp3+ ratio and miR-574-3p/PD-L1 axis in spinal chordoma patients. Cancer Immunol. Immunother..

[20] Zou M.-X., Lv G.-H., Wang X.-B., Huang W., Li J., Jiang Y., She X.-L. (2019). Clinical impact of the immune microenvironment in spinal chordoma: Immunoscore as an independent favorable prognostic factor. Clin. Neurosurg..

[21] Zou M., Pan Y., Huang W., Zhang T., Escobar D., Wang X., Jiang Y., She X., Lv G., Li J. (2020). A four--factor immune risk score signature predicts the clinical outcome of patients with spinal chordoma. Clin. Transl. Med..

[22] Feng Y., Shen J., Gao Y., Liao Y., Côté G., Choy E., Chebib I., Mankin H., Hornicek F., Duan Z. (2015). Expression of programmed cell death ligand 1 (PD-L1) and prevalence of tumor-infiltrating lymphocytes (TILs) in chordoma. Oncotarget.

[23] Mathios D., Ruzevick J., Jackson C.M., Xu H., Shah S.R., Taube J.M., Burger P.C., McCarthy E.F., Quiñones-Hinojosa A., Pardoll E.M. (2015). PD-1, PD-L1, PD-L2 expression in the chordoma microenvironment. J. Neurooncol..

[24] Zhou J., Jiang Y., Zhang H., Chen L., Luo P., Li L., Zhao J., Lv F., Zou D., Zhang Y. (2019). Clinicopathological implications of TIM3+ tumor-infiltrating lymphocytes and the miR-455-5p/Galectin-9 axis in skull base chordoma patients. Cancer Immunol. Immunother..

[25] He G., Liu X., Pan X., Ma Y. (2020). Cytotoxic T lymphocyte antigen-4 (CTLA-4) expression in chordoma and tumor-infiltrating lymphocytes (TILs) predicts prognosis of spinal chordoma. Clin. Transl. Oncol..

[26] Hu W., Yu J., Huang Y., Hu F., Zhang X., Wang Y. (2018). Lymphocyte-related inflammation and immune-based scores predict prognosis of chordoma patients after radical resection. Transl. Oncol..

[27] Gulluoglu S., Tuysuz E.C., Sahin M., Yaltirik C.K., Kuskucu A., Ozkan F., Dalan A.B., Sahin F., Ture U., Bayrak O.F. (2019). The role of TNF-α in chordoma progression and inflammatory pathways. Cell. Oncol..

[28] Drake M.A.S., Michael B.B.T., Wang X.H., Sait S.J., Earp J.C., Jusko W.J., Ferrone S., Wang E.S., Wetzler M. (2006). The effects of anti-high molecular weight-melanoma associated antigen (HMW-MAA) monoclonal antibodies (mAb) against 11q23 positive acute leukemia cells. Blood.

[29] Maciag P.C., Seavey M.M., Pan Z.-K., Ferrone S., Paterson Y. (2008). Cancer immunotherapy targeting the high molecular weight melanoma-associated antigen protein results in a broad antitumor response and reduction of pericytes in the tumor vasculature. Cancer Res..

[30] Schwab J.H., Boland P.J., Agaram N.P., Socci N.D., Guo T., O’Toole G.C., Wang X., Ostroumov E., Hunter C.J., Block J.A. (2009). Chordoma and chondrosarcoma gene profile: Implications for immunotherapy. Cancer Immunol. Immunother..

[31] Schoenfeld A.J., Wang X., Wang Y., Hornicek F.J., Nielsen G.P., Duan Z., Ferrone S., Schwab J.H. (2016). CSPG4 as a prognostic biomarker in chordoma. Spine J..

[32] Showell C., Binder O., Conlon F.L. (2004). T-box genes in early embryogenesis. Dev. Dyn..

[33] Vujovic S., Henderson S., Presneau N., Odell E., Jacques T.S., Tirabosco R., Boshoff C., Flanagan A.M. (2006). Brachyury, a crucial regulator of notochordal development, is a novel biomarker for chordomas. J. Pathol..

[34] Yang X.R., Ng D., Alcorta D., Liebsch N.J., Sheridan E., Li S., Goldstein A.M., Parry D.M., Kelley M.J. (2009). T (brachyury) gene duplication confers major susceptibility to familial chordoma. Nat. Genet..

[35] Pillay N., Plagnol V., Tarpey P.S., Lobo S.B., Presneau N., Szuhai K., Halai D., Berisha F., Cannon S.R., Mead S. (2012). A common single-nucleotide variant in T is strongly associated with chordoma. Nat. Genet..

[36] Sharifnia T., Wawer M.J., Chen T., Huang Q.-Y., Weir B.A., Sizemore A., Lawlor M.A., Goodale A., Cowley G.S., Vazquez F. (2019). Small-molecule targeting of brachyury transcription factor addiction in chordoma. Nat. Med..

[37] Bradner J.E., Hnisz D., Young R.A. (2017). Transcriptional addiction in cancer. Cell.

[38] Magnaghi P., Salom B., Cozzi L., Amboldi N., Ballinari D., Tamborini E., Gasparri F., Montagnoli A., Raddrizzani L., Somaschini A. (2018). Afatinib is a new therapeutic approach in chordoma with a unique ability to target EGFR and brachyury. Mol. Cancer Ther..

[39] Robinson H., McFarlane R.J., Wakeman J.A. (2020). Brachyury: Strategies for drugging an intractable cancer therapeutic target. Trends Cancer.

[40] Donahue R.N., Grenga I., Lepone L., Gulley J.L., Heery C.R., Madan R., Rodell T.C., Schlom J., Farsaci B. (2014). Identification of tumor associated immune responses against brachyury, a transcription factor and driver of EMT, in chordoma patients receiving a yeast-brachyury vaccine (gi-6301). J. Immunother. Cancer.

[41] Wu J., Liang C., Chen M., Su W. (2016). Association between tumor-stroma ratio and prognosis in solid tumor patients: A systematic review and meta-analysis. Oncotarget.

[42] Zou M.-X., Zheng B.-W., Liu F.-S., Wang X.-B., Hu J.-R., Huang W., Dai Z.-H., Zhang Q.-S., Zhong H., Jiang Y. (2019). The relationship between tumor-stroma ratio, the immune microenvironment, and survival in patients with spinal chordoma. Neurosurgery.

[43] Italiano A. (2016). Role of the EZH2 histone methyltransferase as a therapeutic target in cancer. Pharmacol. Ther..

[44] Kim K.H., Kim W., Howard T.P., Vazquez F., Tsherniak A., Wu J.N., Wang W., Haswell J.R., Walensky L.D., Hahn W.C. (2015). SWI/SNF-mutant cancers depend on catalytic and non-catalytic activity of EZH2. Nat. Med..

[45] Antonelli M., Raso A., Mascelli S., Gessi M., Nozza P., Coli A., Gardiman M.P., Arcella A., Massimino M., Buttarelli F.R. (2017). SMARCB1/INI1 involvement in pediatric chordoma. Am. J. Surg. Pathol..

[46] Mobley B.C., McKenney J.K., Bangs C.D., Callahan K., Yeom K.W., Schneppenheim R., Hayden M.G., Cherry A.M., Gokden M., Edwards M.S.B. (2010). Loss of SMARCB1/INI1 expression in poorly differentiated chordomas. Acta Neuropathol..

[47] Onishi A.C., Hincker A.M., Lee F.Y. (2011). Surmounting chemotherapy and radioresistance in chondrosarcoma: Molecular mechanisms and therapeutic targets. Sarcoma.

[48] Simard F.A., Richert I., Vandermoeten A., Decouvelaere A.-V., Michot J.-P., Caux C., Blay J.-Y., Dutour A. (2017). Description of the immune microenvironment of chondrosarcoma and contribution to progression. OncoImmunology.

[49] Kostine M., Cleven A.H., de Miranda N., Italiano A., Cleton-Jansen A.-M., Bovée J.V.M.G. (2016). Analysis of PD-L1, T-cell infiltrate and HLA expression in chondrosarcoma indicates potential for response to immunotherapy specifically in the dedifferentiated subtype. Mod. Pathol..

[50] Richert I., Gomez-Brouchet A., Bouvier C., Pinieux G.D.B.D., Karanian M., Blay J.-Y., Dutour A. (2020). The immune landscape of chondrosarcoma—Potential for therapeutic targeting of CSFR1+ macrophages. J. Bone Oncol..

[51] Stanley E.R., Chitu V. (2014). CSF-1 receptor signaling in myeloid cells. Cold Spring Harb. Perspect. Biol..

[52] Morrissey M.A., Vale R.D. (2019). CD47 suppresses phagocytosis by repositioning SIRPA and preventing integrin activation. bioRxiv.

[53] Castellanos J.R., Purvis I.J., Labak C.M., Guda M.R., Tsung A.J., Velpula K.K., Asuthkar S. (2017). B7-H3 role in the immune landscape of cancer. Am. J. Clin. Exp. Immunol..

[54] Monney L., Sabatos C.A., Gaglia J.L., Ryu A., Waldner H., Chernova T., Manning S., Greenfield E.A., Coyle A.J., Sobel R.A. (2002). Th1-specific cell surface protein Tim-3 regulates macrophage activation and severity of an autoimmune disease. Nat. Cell Biol..

[55] Sakuishi K., Apetoh L., Sullivan J.M., Blazar B.R., Kuchroo V.K., Anderson A.C. (2010). Targeting Tim-3 and PD-1 pathways to reverse T cell exhaustion and restore anti-tumor immunity. J. Exp. Med..

[56] Di Carlo E., Cappello P., Sorrentino C., D’Antuono T., Pellicciotta A., Giovarelli M., Forni G., Musiani P. (2005). Immunological mechanisms elicited at the tumour site by lymphocyte activation gene-3 (LAG-3) versus IL-12: Sharing a common Th1 anti-tumour immune pathway. J. Pathol..

[57] Ikezoe T. (2008). Aurora kinases as an anti-cancer target. Cancer Lett..

[58] Liang X., Wang D., Wang Y., Zhou Z., Zhang J., Li J. (2012). Expression of Aurora Kinase A and B in chondrosarcoma and its relationship with the prognosis. Diagn. Pathol..

[59] Rankin E.B., Giaccia A.J. (2008). The role of hypoxia-inducible factors in tumorigenesis. Cell Death Differ..

[60] Chen C., Ma Q., Ma X., Liu Z., Liu X. (2011). Association of elevated HIF-2α levels with low Beclin 1 expression and poor prognosis in patients with chondrosarcoma. Ann. Surg Oncol..

[61] Chen C., Zhou H., Wei F., Jiang L., Liu X., Liu Z., Ma Q. (2011). Increased levels of hypoxia-inducible factor-1α are associated with Bcl-xL expression, tumor apoptosis, and clinical outcome in chondrosarcoma. J. Orthop. Res..

[62] Yoo B.H., Wu X., Derouet M., Haniff M., Eskelinen E.-L., Rosen K. (2009). Hypoxia-induced downregulation of autophagy mediator Beclin 1 reduces the susceptibility of malignant intestinal epithelial cells to hypoxia-dependent apoptosis. Autophagy.

[63] Karantza-Wadsworth V., White E. (2007). Role of autophagy in breast cancer. Autophagy.

[64] Shen Y., Li D.-D., Wang L.-L., Deng R., Zhu X.-F. (2008). Decreased expression of autophagy-related proteins in malignant epithelial ovarian cancer. Autophagy.

[65] Ahn C.H., Jeong E.G., Lee J.W., Kim M.S., Kim S.H., Yoo N.J., Lee S.H. (2007). Expression of beclin-1, an autophagy-related protein, in gastric and colorectal cancers. APMIS.

[66] Miracco C., Cosci E., Oliveri G., Luzi P., Pacenti L., Monciatti I., Mannucci S., De Nisi M.C., Toscano M., Malagnino V. (2007). Protein and mRNA expression of autophagy gene Beclin 1 in human brain tumours. Int. J. Oncol..

[67] Yan H., Parsons D.W., Jin G., McLendon R., Rasheed B.A., Yuan W., Kos I., Batinic-Haberle I., Jones S., Riggins G.J. (2009). IDH1andIDH2 mutations in gliomas. N. Engl. J. Med..

[68] Mardis E.R., Ding L., Dooling D.J., Larson D.E., McLellan M.D., Chen K., Koboldt D.C., Fulton R.S., Delehaunty K.D., McGrath S.D. (2009). Recurring mutations found by sequencing an acute myeloid leukemia genome. N. Engl. J. Med..

[69] Amary M.F., Bacsi K., Maggiani F., Damato S., Halai D., Berisha F., Pollock R., O’Donnell P., Grigoriadis A., Diss T. (2011). IDH1 and IDH2 mutations are frequent events in central chondrosarcoma and central and periosteal chondromas but not in other mesenchymal tumours. J. Pathol..

[70] Amary M.F., Damato S., Halai D., Eskandarpour M., Berisha F., Bonar F., McCarthy S., Fantin V.R., Straley K.S., Lobo S. (2011). Ollier disease and Maffucci syndrome are caused by somatic mosaic mutations of IDH1 and IDH2. Nat. Genet..

[71] Li L., Paz A.C., Wilky B.A., Johnson B., Galoian K., Rosenberg A., Hu G., Tinoco G., Bodamer O., Trent J.C. (2015). Treatment with a small molecule Mutant IDH1 inhibitor suppresses tumorigenic activity and decreases production of the oncometabolite 2-hydroxyglutarate in human chondrosarcoma cells. PLoS ONE.

[72] Suijker J., Oosting J., Koornneef A., Struys E.A., Salomons G.S., Schaap F.G., Waaijer C.J., Wijers-Koster P.M., Bruijn I.H.B.-D., Haazen L. (2015). Inhibition of mutant IDH1 decreases D-2-HG levels without affecting tumorigenic properties of chondrosarcoma cell lines. Oncotarget.

[73] Rozeman L.B., De Bruijn I.H.B., Bacchini P., Staals E.L., Bertoni F., Bovée J.V.M.G., Hogendoorn P.C. (2009). Dedifferentiated peripheral chondrosarcomas: Regulation of EXT-downstream molecules and differentiation-related genes. Mod. Pathol..

[74] Tiet T.D., Hopyan S., Nadesan P., Gokgoz N., Poon R., Lin A.C., Yan T., Andrulis I.L., Alman B.A., Wunder J.S. (2006). Constitutive Hedgehog signaling in chondrosarcoma up-regulates tumor cell proliferation. Am. J. Pathol..

[75] Zou Z., Tao T., Li H., Zhu X. (2020). mTOR signaling pathway and mTOR inhibitors in cancer: Progress and challenges. Cell Biosci..

[76] Perez J., Decouvelaere A.V., Pointecouteau T., Pissaloux D., Michot J.P., Besse A., Blay J.Y., Dutour A. (2012). Inhibition of chondrosarcoma growth by mTOR inhibitor in an in vivo syngeneic rat model. PLoS ONE.

[77] Addie R.D., De Jong Y., Alberti G., Kruisselbrink A.B., Que I., Baelde H., Bovée J.V. (2019). Exploration of the chondrosarcoma metabolome; the mTOR pathway as an important pro-survival pathway. J. Bone Oncol..

[78] Migliorini D., Mach N., Aguiar D., Vernet R., Landis B.N., Becker M., McKee T., Dutoit V., Dietrich P.-Y. (2017). First report of clinical responses to immunotherapy in 3 relapsing cases of chordoma after failure of standard therapies. OncoImmunology.

[79] Mach N., Vernet R., Belkouch M.-C., Luy P., Ancrenaz V., Teta P., Blazek N., Grandjean N., Wasem J., Grogg J. (2016). MVX-ONCO-1 phase 1 final results of the first personalized cell-based immunotherapy using cell encapsulation technology. Ann. Oncol..

[80] (2020). Maxivas. MVX-ONCO-1 in Patients with Solid Tumor.

[81] Wu X., Lin X., Chen Y., Kong W., Xu J., Yu Z. (2020). Response of metastatic chordoma to the immune checkpoint inhibitor pembrolizumab: A case report. Front. Oncol..

[82] Scognamiglio G., De Chiara A., Parafioriti A., Armiraglio E., Fazioli F., Gallo M., Aversa L., Camerlingo R., Cacciatore F., Colella G. (2019). Patient-derived organoids as a potential model to predict response to PD-1/PD-L1 checkpoint inhibitors. Br. J. Cancer..

[83] Fujii R., Friedman E.R., Richards J., Tsang K.Y., Heery C.R., Schlom J., Hodge J.W. (2016). Enhanced killing of chordoma cells by antibody-dependent cell-mediated cytotoxicity employing the novel anti-PD-L1 antibody avelumab. Oncotarget.

[84] Hamilton D.H., Litzinger M.T., Jales A., Huang B., Fernando R.I., Hodge J.W., Ardiani A., Apelian D., Schlom J., Palena C. (2013). Immunological targeting of tumor cells undergoing an epithelial-mesenchymal transition via a recombinant brachyury-yeast vaccine. Oncotarget.

[85] Heery C.R., Singh B.H., Rauckhorst M., Marté J.L., Donahue R.N., Grenga I., Rodell T.C., Dahut W.L., Arlen P.M., Madan R.A. (2015). Phase I trial of a yeast-based therapeutic cancer vaccine (GI-6301) targeting the transcription factor brachyury. Cancer Immunol. Res..

[86] DeMaria P.J., Bilusic M., Park D., Heery C.R., Madan R.A., Strauss J., Donahue R.N., Marte J., Gilbert M.R., Steinberg S.M. (2020). A randomized, double-blind, phase II clinical trial of GI-6301 (yeast-brachyury vaccine) versus placebo in combination with standard of care definitive radiotherapy in locally advanced, unresectable, chordoma. J. Clin. Oncol..

[87] Gounder M.M., Zhu G., Roshal L., Lis E., Daigle S.R., Blakemore S.J., Michaud N.R., Hameed M., Hollmann T.J. (2019). Immunologic correlates of the abscopal effect in a SMARCB1/INI1-negative poorly differentiated chordoma after EZH2 inhibition and radiotherapy. Clin. Cancer Res..

[88] Italiano A., Soria J.C., Toulmonde M., Michot J.M., Lucchesi C., Varga A., Coindre J.M., Blakemore S.J., Clawson A., Suttle B. (2018). Tazemetostat, an EZH2 inhibitor, in relapsed or refractory B-cell non-Hodgkin lymphoma and advanced solid tumours: A first-in-human, open-label, phase 1 study. Lancet Oncol..

[89] (2021). Sidney Kimmel Comprehensive Cancer Center. Nivolumab with or without Stereotactic Radiosurgery in Treating Patients with Recurrent, Advanced, or Metastatic Chordoma. https://clinicaltrials.gov/ct2/show/NCT02989636.

[90] (2020). Jonsson Comprehensive Cancer Center. Nivolumab and Relatlimab in Treating Participants with Advanced Chordoma. https://clinicaltrials.gov/ct2/show/NCT03623854.

[91] Paoluzzi L., Cacavio A., Ghesani M., Karambelkar A., Rapkiewicz A., Weber J., Rosen G. (2016). Response to anti-PD1 therapy with nivolumab in metastatic sarcomas. Clin. Sarcoma Res..

[92] Wagner M.J., Ricciotti R.W., Mantilla J., Loggers E.T., Pollack S.M., Cranmer L.D. (2018). Response to PD1 inhibition in conventional chondrosarcoma. J. Immunother. Cancer.

[93] Tawbi H.A., Burgess M., Bolejack V., Van Tine B.A., Schuetze S.M., Hu J., D’angelo S., Attia S., Riedel S.F., Priebat D.A. (2017). Pembrolizumab in advanced soft-tissue sarcoma and bone sarcoma (SARC028): A multicentre, two-cohort, single-arm, open-label, phase 2 trial. Lancet Oncol..

[94] Katz D., Assaf-Harofeh Medical Center (2017). A Phase II of Nivolumab Plus Ipilimumab in Non-Resectable Sarcoma and Endometrial Carcinoma. https://clinicaltrials.gov/ct2/show/NCT02982486.

[95] (2021). Sarcoma Oncology Research Center. Nivolumab (Opdivo®) Plus ABI-009 (Nab-rapamycin) for Advanced Sarcoma and Certain Cancers. https://clinicaltrials.gov/ct2/show/NCT03190174.

[96] (2021). Agios Pharmaceuticals. Study of Orally Administered AG-120 in Subjects with Advanced Solid Tumors, Including Glioma, with an IDH1 Mutation. https://clinicaltrials.gov/ct2/show/NCT02073994.

[97] (2021). Bayer. Phase I Study of BAY1436032 in IDH1-mutant Advanced Solid Tumors. https://clinicaltrials.gov/ct2/show/NCT02746081.

[98] (2021). Agios Pharmaceuticals. Study of Orally Administered AG-221 in Subjects with Advanced Solid Tumors, Including Glioma, and with Angioimmunoblastic T-cell Lymphoma, with an IDH2 Mutation Subjects with Advanced Solid Tumors, Including Glioma, and with Angioimmunoblastic T-Cell Lymp. https://clinicaltrials.gov/ct2/show/results/NCT02073994.

[99] (2021). National Cancer Institute. Vismodegib in Treating Patients with Advanced Chondrosarcomas. https://clinicaltrials.gov/ct2/show/NCT01267955.

